# Exploring the Ethical Implications of ChatGPT in Medical Education: Privacy, Accuracy, and Professional Integrity in a Cross-Sectional Survey

**DOI:** 10.7759/cureus.75895

**Published:** 2024-12-17

**Authors:** Hafiz Muhammad Amad Abdullah, Noor-i-Kiran Naeem, Ghufran Ali Malkana, Masib Javed, Malik Muhammad Shahzaib

**Affiliations:** 1 Medical Education, ABWA Medical College, Faisalabad, PAK; 2 Obstetrics, Gynecology and Infertility, Dr Rehmatullah General And Eye Hospital, Gojra, PAK; 3 Infertility, Australian Concept Infertility Centre, Faisalabad, PAK

**Keywords:** accuracy, artificial intelligence, chatgpt, critical thinking, ethical concerns, medical education, privacy, professional integrity

## Abstract

Background: The inclusion of artificial intelligence in medical education, specifically through the use of ChatGPT (OpenAI, San Francisco, CA), has transformed learning and generated many ethical questions. This study aims to analyze the medical students' ethical concerns about using ChatGPT in medical education, focusing on privacy, accuracy, and professional integrity.

Methods: The study format was a cross-sectional survey distributed to 219 medical students at ABWA Medical College, Pakistan. A pre-validated, pre-structured questionnaire was created with Google Forms, including questions regarding the accuracy of ChatGPT, confidentiality, and its impact on the students' critical thinking faculties. This information was collected after obtaining informed consent. The data collected were descriptively and inferentially analyzed using SPSS software version 26.0 (IBM Corp., Armonk, NY).

Results: In total, 95% of respondents (n = 190) confirmed that the information provided by ChatGPT was accurate, and 80% (n = 160) stated that they trusted the medical information from the tool. However, 83% (n = 166 students) indicated concerns about privacy and data security. While 69% of participants (n = 138) discovered that ChatGPT supplemented their critical thinking skills, the rest (31%; n = 62) believed it led to decreased autonomy over time. However, since health science-related courses often involve sensitive patient information, 22% (n = 44) of students raised concerns about using ChatGPT in future medical education due to the potential issues with privacy and the risk of inaccuracies in recorded information.

Conclusion: ChatGPT offers promising educational benefits in medical training but raises significant ethical concerns, particularly regarding data privacy and the potential for over-reliance. The results suggest the need for responsible integration of AI in medical education, ensuring it supplements rather than replaces traditional learning methods.

## Introduction

Technology has left no domain untouched; even medical education has been impacted drastically due to the advancement in several domains. In some studies, the introduction of advanced artificial intelligence tools such as ChatGPT (OpenAI, San Francisco, CA) within medical education has been considered a key development [1]. Consequently, this paper aims to analyze these ethical implications, emphasizing privacy, factuality, and a sense of professionalism.

With time, ChatGPT could become a potent educational tool because of its potential to "speak" in a teachable language and is considered by some to be an outstanding learning environment [2]. It facilitates medical education by reproducing a patient interface, enabling learners to engage in specific clinical inquiries, and closing the gap between theory and background learning processes. However, as ChatGPT was rolled out for healthcare delivery, it presented many ethical challenges. This aligns with a general feeling among many healthcare professionals that ChatGPT contains most of this information [2-3]. Additionally, using ChatGPT judiciously is vital; misinformation or inaccuracies could harm patient health and threaten safety, security, and privacy rights, thus necessitating the requirement for protective measures [3]. Furthermore, a possible hypothesis is that students will become dependent on ChatGPT and therefore lose a degree of control over their cognitive processes [4].

This study aims to outline the ethical considerations of using ChatGPT in medical education, focusing on risks and providing a roadmap for ethical use. Ensuring privacy, accuracy, and integrity can enhance learning while minimizing potential harm.

## Materials and methods

We performed a cross-sectional survey at ABWA Medical College, Pakistan, to analyse the ethical concerns of ChatGPT among MBBS students. Ethical clearance for the study was obtained from the ABWA Medical College Institutional Review Committee via clearance form no. 986/2024. This study was conducted in a timeframe of around five to six months, starting in June 2024 and completing in October 2024. This questionnaire received 219 responses, indicating several different academic years in college. The students developed the questions and even provided extensive descriptions of study characteristics and correct responses. Informed consent was given by students. Applying the standard techniques to calculate an appropriate survey sample size gave us our number of respondents for this research. We assumed that the outcome factor would happen 50% of the time in the population, with a margin error of +5%. This analysis utilized a 95% confidence interval.

A custom questionnaire was developed using Google Forms to assess ethical concerns related to ChatGPT, focusing on privacy, accuracy, and professional integrity. The questionnaire was designed by the authors, reviewed by the supervisor and coauthors, and further evaluated and approved by the ethical review committee of ABWA Medical College. The questionnaire was pilot-tested with a small group of participants to refine the final version. These efforts contribute to the questionnaire’s reliability, though formal validation was not performed.

Data analysis was accomplished with the SPSS version 26.0 program (IBM Corp., Armonk, NY). The descriptive statistics provided the student perceptions regarding the ethical implications of ChatGPT.

## Results

The online questionnaire was prepared, and we enlisted 219 medical students as a sample for the survey. A total of 91.32% (200/219) completed questionnaires were returned. Most of the participants, around 55% (n = 101), were 21 to 23 years of age with a mean age of 22.48 years (standard deviation (SD) of <0.56). The cohort consisted of all students enrolled in the MBBS program at the college who readily agreed to participate in the project. Fourth-year students constituted the largest group at 28.5%, followed closely by second-year students at 25.5%. The smallest group to participate comprised the students from the fifth year, constituting only 12% of the total respondents. The remaining proportions were 20.5% and 13.5% among students in their first and third years of study, respectively.

The demographic characteristics of those in the main analysis cohort are shown in Table 1. The gender breakdown was roughly even, with 53% male and 47% female. The following comprehensive list of cumulative statistics provides context to the demographics of the participants in this study before we examine their perceptions and experiences with ChatGPT in the findings (Table 1).

**Table 1:** Demographic characteristics of study participants in the cross-sectional survey

Demographic Characteristics	Study Participants (n=200)	Percentage (%)
Gender		
Male	106	53
Female	94	47
Age (in years)		
18-20	56	28
21-23	110	55
24-26	34	17
MBBS students		
First-year students	41	20.5
Second-year students	51	25.5
Third-year students	27	13.5
Fourth-year students	57	28.5
Fifth-year students	24	12

**Figure 1:**
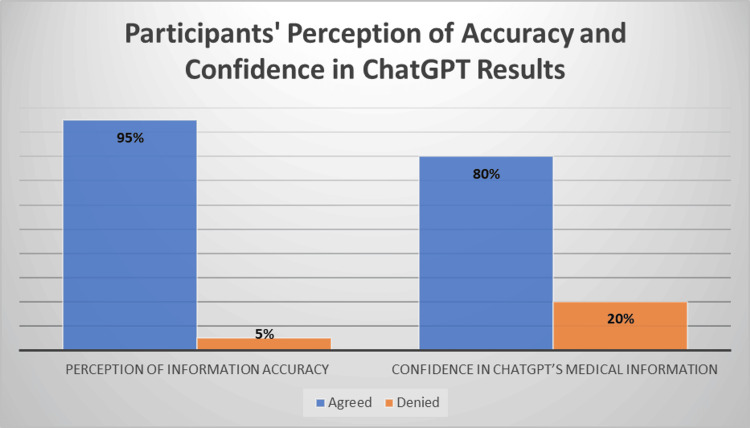
Participants' perception of accuracy and confidence in ChatGPT results

When evaluating the accuracy of the information ChatGPT shared with participants, 190 students (95% of the total population) agreed that the information provided by ChatGPT was accurate, and only 10 students (5% of the total population) did not feel that the information on ChatGPT is correct, as presented in Figure 1. Respondents were also asked about their confidence in the medical information provided by ChatGPT. Regarding medical information confidence, 160 students (approximately 80%) indicated that ChatGPT only provided accurate medical details in some cases. The right panel in Figure 1 shows that 40 students, representing 20% of the total population, answered "no."

**Figure 2:**
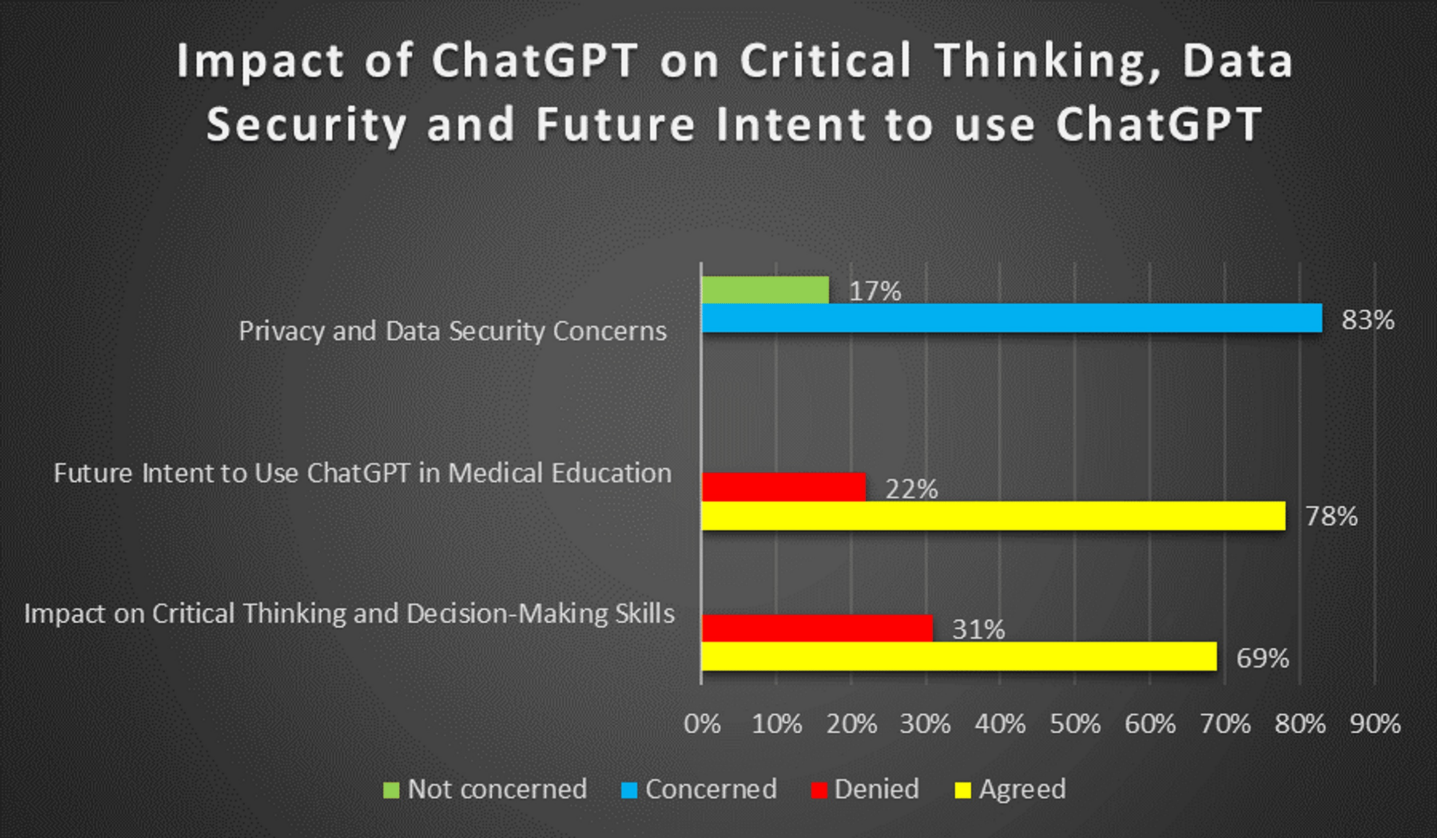
Bar chart showing the concerns about decline in critical thinking ability, data security and future intent to use ChatGPT

During the survey, we also enquired about how worried users were regarding their privacy and confidentiality when using ChatGPT. When questioned, approximately 166 students, nearly 83% of the overall study population, had privacy and data confidentiality-related concerns, however, some outliers stated that there was no issue in such situations, which consists of only 34 students, i.e., 17%, as shown in Figure 2.

ChatGPT’s effect on critical thinking and decision-making skills was evaluated. A total of 138 students (69% of the total population) declared that ChatGPT improved their critical thinking skills; on the other hand, 62 students (31% of the total population) declared that it reduced their skills (as illustrated in Figure 2). Finally, at the end of our presentation, we asked them about their future considerations of using ChatGPT in medical education for a scale model. Of the total population, around 156 students (78%) were in favor of using ChatGPT for medical education, and approximately 44 students, representing 22% of the overall, opposed our hypothesis as shown in Figure 2.

## Discussion

In the present study, the cross-sectional survey of medical students revealed how they perceive ChatGPT's accuracy in its activity, build trust among users, maintain privacy, and affect critical thinking skills during this study.

A total of 200 students were included in the study, representing a broad range of medical student classes and genders, providing methodological diversity for analysis in relation to ChatGPT. This approach is crucial as it allows our findings to be narrowed down more easily across the population of medical students, ensuring a wide range of opinions. The study exhibited a strong response rate of 91.32%, which not only verified the accuracy of the data provided by ChatGPT but also demonstrated that students were intrigued by the opportunity to gather data from it.

In previous reports that have cited the reliability of AI-powered technologies in universities, almost 95% of students confirmed ChatGPT's information [5,6]. Nevertheless, 5% of the students reported that ChatGPT generated mistakes, highlighting the need for real-time examination and verification of the information provided. This finding stresses the importance of critical validation before deploying AI, as even a small doubt can create significant issues, especially in a field like medicine [7].

When they saw the medical information given to them from ChatGPT, 80% of students stated that they were confident about the information provided. This result is consistent with the recent data, which shows AI to be a promising new educational modality in medicine that can produce better results if applied properly [8]. Nonetheless, the 20% unsure responses for medical information on ChatGPT reflect a need to improve upon how effective and reliable this tool can really be. Therefore, the lowering scores of some students may stem from misusing this software [8]. Instead of only relying on AI for educational answers, they should maintain a healthy skepticism towards this technology and apply this mindset in their work as clinicians, physicians, or medical students [9].

Regarding privacy and data security, 83% of students had concerns regarding the privacy of their data. This correlates with the other studies; among the general issues associated with the ethical challenges of AI in education, the most mentioned is privacy [10-12]. Security and obvious transparency around how data is handled in AI tools such as ChatGPT are necessary to prevent data breaches. To solve this problem, we need to build trust and confidence in students in their interactions with these technologies [13].

In our study, the majority of students stated that ChatGPT played an important part in strengthening their thought processes and decision-making abilities. A total of 69% of students said ChatGPT ended up improving their critical thinking skills; only 31% reported that it weakened such faculties. The conclusion may be seen as ironic, given that AI, which is intended to support the development of critical thinking with quick access to information and availability of multiple points of view, might lead even further away from independent thought or problem-solving [14]. This aligns with literature that has raised concerns that AI could contribute to individuals becoming dependent learners, to the detriment of the learning process [15, 16].

Finally, a total of 78% of participants indicated they would make better use of ChatGPT for their medical studies in their future activities. Nonetheless, 22% of the participants indicated skepticism or refusal to use ChatGPT should this technology be implemented in the future due to concerns about accuracy, privacy, and the effect that might have on critical thinking. In this regard, we consider ChatGPT as an advancement in the field; however, it must also be embraced cautiously within medical education to manage sensitive concepts such that technology serves as a supplement rather than a substitution for traditional methods of learning [16-18].

Limitations

This study is limited by the use of self-reported data, which may introduce bias. It was conducted at a single medical college, limiting generalizability to other settings. The cross-sectional design provides a snapshot in time and may overlook more nuanced ethical dilemmas or long-term impacts of ChatGPT on medical education. The study did not assess long-term outcomes, and future research should explore diverse populations using longitudinal approaches and mixed methods to gain a more comprehensive understanding of ChatGPT’s effects.

## Conclusions

We consider the results of this study to have important implications for presenting and employing ChatGPT in a medical education context. Privacy issues will have to be addressed first and foremost. Selection bias also remains a problem. Moreover, these developments highlight the importance of ongoing evaluation and improvement of tools like this to ensure they support the next generation of competent, independent clinicians.

## Appendices

Sample Questionnaire

1. Age:

a. 18-20 Years

b. 21-23 Years

c. 24-26 Years

2. Gender:

a. Male

b. Female

3. Year of Study:

a. First year

b. Second year

c. Third year

d. Fourth year

e. Fifth year

4. How would you rate the accuracy of the information provided by ChatGPT?

a. Accurate

b. Inaccurate

5. How confident are you in the medical information provided by ChatGPT?

a. Confident

b. Not confident

6. How concerned are you about the privacy and confidentiality of your data when using ChatGPT?

a. Concerned

b. Not concerned

7. Do you think using ChatGPT could impact your critical thinking skills and compromise the development of your autonomous decision-making skills?

a. Agree

b. Disagree

8. If given the choice, would you continue to use ChatGPT for medical education in your future academic years?

a. Yes

b. No
